# Supplementary material for: Optimization of a Lambda-RED Recombination Method for Rapid Gene Deletion in Human Cytomegalovirus
Source: Int J Mol Sci. 2021 Sep 29;22(19):10558. doi: 10.3390/ijms221910558 (PMC8508972; doi:10.3390/ijms221910558)

**Supplementary Material**

**Table S1.** Predicted size (bp) of the PCR products using the different oligonucleotides combination for both WT and mutant template genome for all the assayed genes.

| Gene | WT ab[1] | Mutant ab[1] | Mutant ac[2] | Mutant db[3] |
|---|---|---|---|---|
| **UL2** | 520 | 1961 | 1067 | 1285 |
| **UL5** | 928 | 2151 | 1079 | 1363 |
| **UL6** | 1342 | 2211 | 1226 | 1276 |
| **UL7** | 1114 | 2169 | 1141 | 1319 |
| **UL8** | 831 | 2186 | 1206 | 1291 |
| **UL9** | 1157 | 2194 | 1179 | 1306 |
| **UL44** | 1903 | 2325 | 1454 | 1162 |
| **UL121** | 893 | 2125 | 1179 | 1237 |
| **US7** | 1030 | 2081 | 1070 | 1302 |
| **US9** | 1192 | 2172 | 1050 | 1413 |
| **US13** | 1150 | 2668 | 1130 | 1249 |
| **US14** | 1387 | 2178 | 1119 | 1350 |
| **US16** | 1549 | 2343 | 1425 | 1209 |
| **US17** | 1410 | 2252 | 1239 | 1304 |
| **US18** | 1362 | 2261 | 1208 | 1344 |
| **US20** | 1319 | 2278 | 1189 | 1380 |
| **US21** | 1242 | 2246 | 1270 | 1267 |
| **US30** | 1974 | 2648 | 1310 | 1629 |
| **pp65** | 1905 | 1949 | 1044 | 1196 |
| **IE1** | 2132 | 2096 | 1018 | 1369 |
| **gO** | 1796 | 2119 | 1131 | 1279 |
| **gM** | 1410 | 2015 | 1126 | 1180 |

[1] Amplification using the primers upstream and downstream (ab) of the target gene using the wild-type BAC DNA (WT) as a template.
[2] Amplification using the primer upstream of the deleted gene and an internal Kan primer (ac).
[3] Amplification using the internal Kan primer and a primer downstream of the deleted gene (db).

**Figure S1. Representative step-by-step process to validate UL6 deletion mutant.** **(A)** DNA of the WT BAC and the BAC obtained from 8 colonies were digested with *Eco*RI and *Bam*HI enzymes and the restriction patterns obtained are shown. Digestion products were separated by electrophoresis on a 1% agarose gel. Lane 1: wild-type BAC DNA (WT). Lane 2-9: BAC DNA obtained from the 8 tested colonies for ΔUL6. **(B)** To test the insertion of the Kan cassette in the UL6 ORF, the BACs obtained from the 8 tested colonies were used for PCR amplification using the upstream and dowstream (ab) primers. Only clones 3 and 4 (highlighted in green) were positive for the insertion, while the band obtained from the other 6 clones had the size expected for WT. **(C)** A second PCR amplification was performed using clones 3 and 4 using the internal Kan primer and a primer downstream or upstream of the deleted gene (db or ac, respectively). PCR products were sequenced. For all the experiments 1 kb DNA molecular weight marker was used as a ladder.

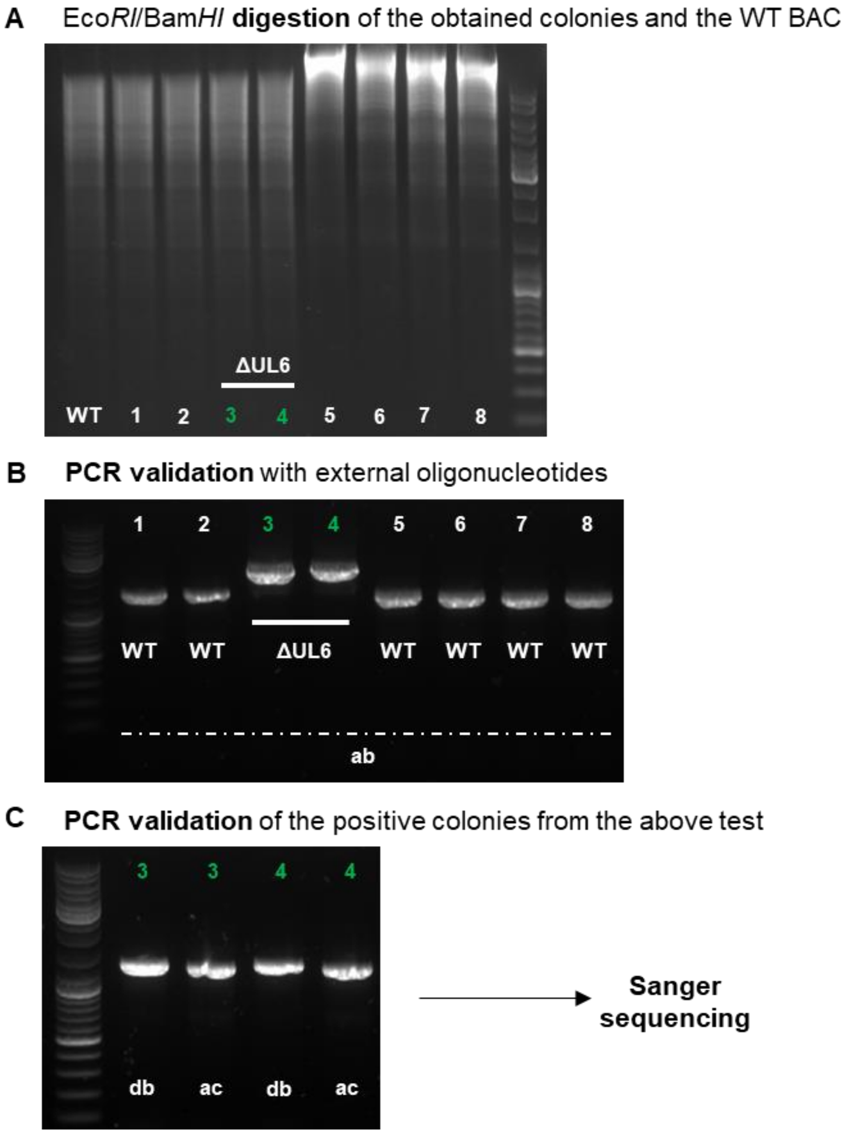

**Figure S2.** Representative images of PCR amplification products obtained to confirm the gene deletion of the indicated ORF. PCR products were separated by electrophoresis on a 1.5% agarose gel. Lane 1: 1 kb DNA molecular weight marker used as a ladder. Lane 2 and 3: PCR amplification products obtained using the primers upstream and downstream (ab) of the target gene using as a template either the wild-type BAC DNA (WT) or the indicated deletion mutant BAC DNA. Lane 4: PCR amplification product obtained using the internal Kan primer and a primer downstream of the deleted gene (db). Lane 5: PCR amplification product obtained using the primer upstream of the deleted gene and an internal Kan primer (ac).

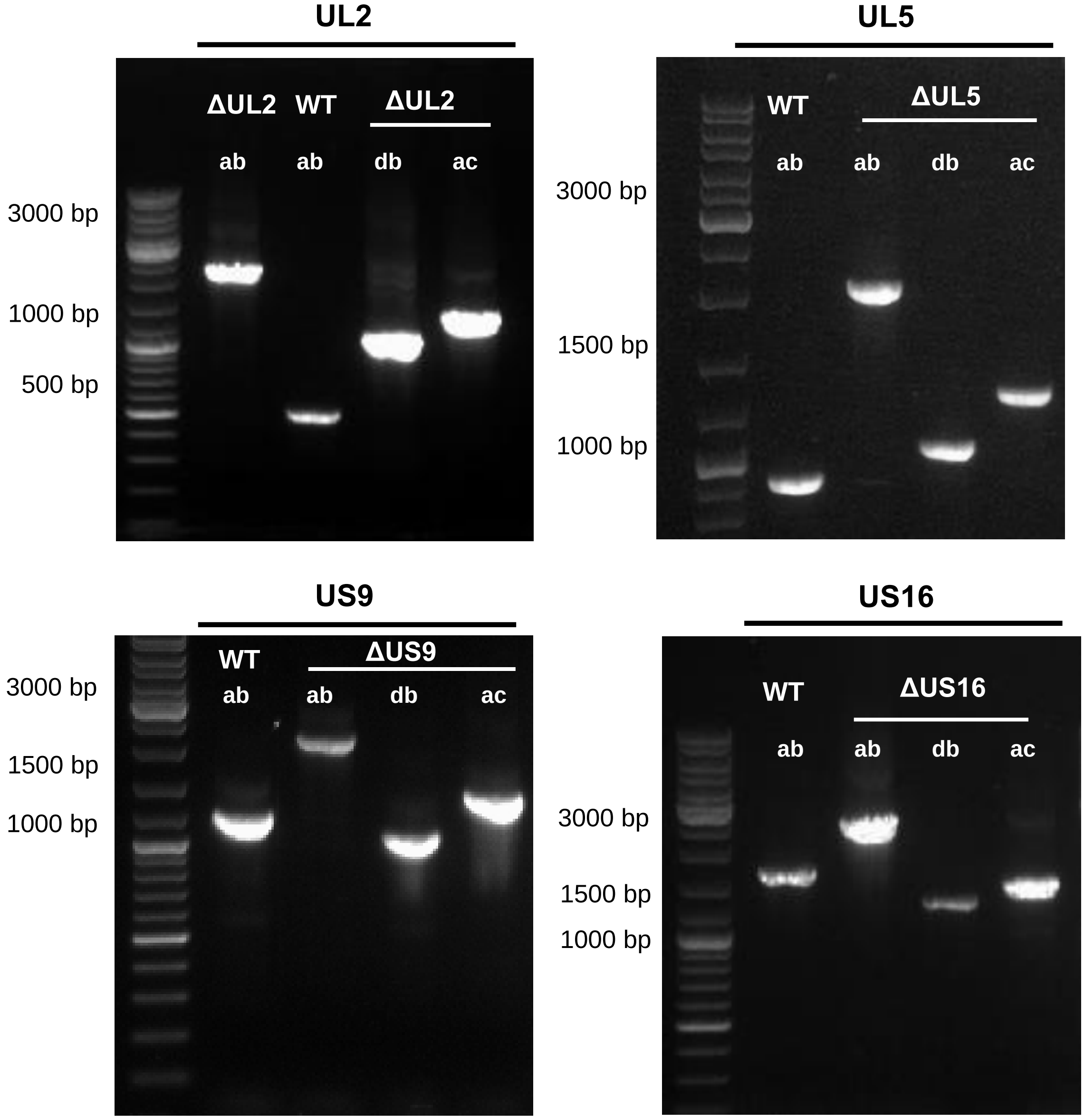

Supplement: Supplementary file 1 [file ijms-22-10558-s001.zip › ijms-1368796-supplementary.pdf]
